# Benefit incidence analysis in public health facilities in India: utilization and benefits at the national and state levels

**DOI:** 10.1186/s12939-019-0921-6

**Published:** 2019-01-21

**Authors:** Diana Bowser, Bryan Patenaude, Manjiri Bhawalkar, Denizhan Duran, Peter Berman

**Affiliations:** 1 Heller School for Social Policy and Management, Brandeis University, 415 South Street, Waltham, MA 02453 USA; 2 Johns Hopkins Bloomberg School of Public Health, Baltimore, USA; 3 Harvard University T.H. Chan School of Public Health, Boston, USA

**Keywords:** Health financing, Inequality, International health, Resource allocation, Health care utilization, India

## Abstract

**Background:**

Benefit Incidence Analysis (BIA) is used to understand the distribution of health care utilization and spending in comparison to income distribution. The results can illustrate how effectively governments allocate limited resources towards meeting the needs of the poor. In analyzing the distribution of public spending on inpatient, outpatient, and deliveries, this paper represents the most recent BIA completed in India.

**Methods:**

In order to conduct the BIA statistical analysis for this project, 2014 utilization data from the most recently completed Indian National Sample Survey (NSS) was used. Unit costs were estimated for primary care, hospital inpatient, hospital outpatient, and deliveries. Concentration curves and concentration indices were estimated both at the national and state levels. Analyses were reported for overall utilization, as well as for the gross and net benefits for inpatient, outpatient, and deliveries.

**Results:**

According to the results, utilization of government inpatient and delivery services is pro-poor. When gross and net benefits are included in the analysis, services become more equal and less pro-poor. Gross benefits, which are measured with state-level unit costs, are virtually equal for all services. Although there are some pro-poor gross benefits trends for national outpatient services, the results also show that the equality of national gross benefits trends hides a significant disparity across Indian States. While a number of Indian States have outpatient gross benefits that are pro-poor, few show pro-poor benefits for inpatient and delivery services. Net benefits, which considers both unit costs for each respective service, and out-of-pocket (OOP) expenditures, trend similarly to gross benefits. In addition, those who use public facilities spend considerable OOP to supplement government services.

**Conclusions:**

This BIA reveals that government spending on public health care has not resulted in significantly pro-poor services. While some progress has been made relative to deliveries and outpatient services, inpatient stays are not pro-poor. In addition, national results mask significant disparities across Indian states.

**Electronic supplementary material:**

The online version of this article (10.1186/s12939-019-0921-6) contains supplementary material, which is available to authorized users.

## Background

While India has made great strides in many of its national indicators over the last several decades, it still lags behind in key measues of equality. For instance, India’s maternal mortality ratio fell from 556 maternal deaths per 100,000 live births in 1990, to 174 maternal deaths per 100,000 live births in 2015 [1]. Similarly, over this same time period, the national infant mortality rate fell from 88 infant deaths per 1000 live births, to 37.9 infant death per 1000 live births. Despite these national trends, inequality across Indian states varies considerably with an almost five-fold difference in maternal mortality between Assam and Kerala [2]. Furthermore, inequality in regard to financing is also a pressing issue. India has one of the highest levels of Out-of-pocket (OOP) expenditure in the world, at 62.4% of total health expenditures as of 2014 [1]. In addition, a large portion of the population uses the private sector for healthcare, adding to the inequality both in terms of access to care, as well as payment levels.

Benefit Incidence Analysis (BIA) is a well-known method used to understand, quantitatively, the distribution of both health care utilization and spending on health care services in comparison to socioeconomic welfare distribution [3–5]. Unlike simply comparing descriptive statistics by stratified variables, BIA condenses the distribution of benefits over the population into a single number, similar to a gini-coefficient, which can be used to compare results across time and location. Due to this property, BIA has been used in a number of developing settings including Nigeria, Vietnam, Pakistan and Jordan to provide quantitative evidence of how health care services, as well as their accompanying costs, are allocated to different members of the population based on their socioeconomic status [3–8]. BIA has also been applied at the sub-national level in India to address specific questions of equity relating to public health or child delivery care utilization [6, 9].

Understanding BIA in the public sector has clear importance in India at the national level where a large portion of the population relies on public-sector subsidies for basic primary health care as a means to achieve the health-related Sustainable Development Goals and reduce inequality. Specifically, our analysis sheds light on India’s efforts toward achieving several of the SDG targets related to universal health coverage and catastrophic health payments [10]. In addition, the quantification of the distribution of public investiments and out-of-pocket expenditure through benefit incidence analysis in India displays how well coverage and benefits accrue to the most disadvantaged (poorest) populations compared with wealtheir populations as well as where the burden of household expenditures on health are the greatest.

While there have been a number of BIA’s done in countries around the world, the results reported below are the most recent BIA results for India, as well as the most comprehensive in terms of coverage of Indian states [5–8]. A previous BIA was done in India that used the 52nd round of the National Sample Survey with data from 1995 to 1996 in order to examine the provision of public subsidies for various primary care services and inpatient care in 16 Indian States [11]. The results of this analysis showed high levels of distributional inequality for both inpatient and outpatient care, especially in public hospitals. Similar research also showed how national level results can mask some of the lower level variation among utilization rates. For instance, while research in Pakistan found public spending on certain health services to be largely pro-poor, Malik and Ashraf’s analysis also found that the utilization of some services, such as post-natal consultation, and institutional maternal delivery, by the bottom shares of income groups to be pro-rich, again indicating levels of distributional inequality across services [4]. While some BIA studies focus on public subsidies in developed countries specifically, many others provide overviews of subsidies, public investments, or policies in developing settings [12, 13]. Other research employs systematic review to compare the results and application of BIA across settings [5]. The BIA conducted in this paper adds to this body of knowledge, by both, updating and expanding upon India’s BIA analyses from 1995 to 1996 to determine how health policy since this time has impacted the distribution of utilization, benefits, and out-of-pocket expenditure, as well as to contribute to the literature on similar patterns of resource distribution across Asia [3–5].

## Methods

### Data sources

The main data source for this analysis was the 71st round of the Indian National Sample Survey (NSS), conducted in 2014. From this survey, we used a healthcare utilization and expenditure model, which provided both individual and household-level use and expenditure. The survey was a representative sample of 65,932 households and 340,737 individuals with survey weights used to scale up to the national population. The data used from this survey were extracted directly and delineated by health facility type, and included fees paid OOP for inpatient stays, outpatient visits, and deliveries; utilization of inpatient stays, outpatient visits, and delivery utilization; as well as insurance reimbursements for inpatient stays, primary health care visits, and deliveries. All data were gathered in Indian Rupees (INR). Deliveries refer to child births which may be home deliveries or at primary or seconday facilities. Deliveries were separated at the national level due the prioritization of policy attempting to reduce maternal mortality through incentivizing facility-based delivery in India [2, 6].

Government health care expenditures were obtained from Indian state budget documents and Demand for Grants (a more complete explanation of how these expenditure aggregates were estimated can be found in Berman et al. 2017) [14]. Combining these data produced the average government expenditure costs for public care by service type and level of facility. Additionally, separation of primary health care and hence direct unit cost calculations by level of facility could only be calculated for for 16 Indian states (Andhra Pradesh, Assam, Bihar, Chhattisgarh, Gujarat, Jharkhand, Karnataka, Kerala, Madhya Pradesh, Maharashtra, Odisha, Punjab, Rajasthan, Tamil Nadu, Uttar Pradesh, West Bengal). For the remaining 13 Indian States where direct unit cost calculations were not possible, the mean unit costs for the above 16 Indian states were applied.

### Measuring health service utilization

Outpatient visits over a two-week period were reported in the NSS and then annualized to reflect yearly outpatient utilization. Inpatient stays and delivery visits were recorded on a yearly basis in the NSS. Annualized outpatient visits were calculated by multiplying the reported number of outpatient visits in the last 2 weeks by 262. Inpatient stays, outpatient visits, and delivery utilization were weighted by the NSS’s survey weights, which scaled up the individual utilization within each state to be representative of the national level. Both inpatient visits, as well as outpatient visits, included those visits reported at each of three levels of care: Level 1 (Health Sub Centre), Level 2 (PHC, dispensary/Community Health Centre/mobile medical units), and Level 3 (public hospital).

### Unit cost estimates

For all states in India, unit costs were estimated for the different levels of care for both inpatient and outpatient, including primary care, inpatient hospital care, and outpatient hospital care. Unit cost estimates were calculated by dividing state-level government expenditure on primary care and hospital care by the reported utilization of the whole population, by level of primary and hospital care according to the NSS.

### Estimating benefits received

To estimate the value of services received from government facilities, unit costs for both the type of service and level of care were combined with utilization volumes for those services for varying socio-economic groups. Unit costs varied by the level of care used by individuals. In both inpatient and outpatient settings, these cost differences represented different utilization patterns that were then reflected in the BIA analysis. For example, an individual who reported visiting a hospital for outpatient care was allocated a different unit cost than one who reported using a dispensary for an outpatient visit. Additionally, unit costs for the different levels of care were also applied to locations where a woman reported delivering.

Unit costs were defined by the gross benefits for each unit of service received. This represented the governmental cost of each individual unit of health care consumption for each level and type of care. Net benefits were defined as the unit cost minus the OOP expenditure for that service plus the insurance reimbursement for that service. The insurance reimbursement was considered in the calculation because there were a small percent of the sample who received reimbursement directly from through their insurance scheme.

There was a subset of individuals (11% of the total sample) whose reported reimbursements were larger than the unit cost minus the OOP expenditure for that services. This was expected in some cases as the unit-cost calculation was done at the state level, while OOP expenditures were self-reported at the individual level; allowing for some senarios where the individual level OOP expenditure was greater than the state level average unit cost. Furthermore, there was an even smaller cohort of individuals whose self-recalled reimbursements were greater than the self-reported OOP expenditures. For individuals from either of these scenarios, a zero value replaced the negative value.

### Measuring out-of-pocket health spending

OOP spending associated with public facilities was collected both for outpatient visits made to those facilities during the 2 weeks preceding the survey, as well as inpatient and delivery visits for 12 months preceding the survey. As was the case for health utilization, OOP spending on outpatient care was annualized. However, OOP spending for outpatient, inpatient, and delivery were weighted by the NSS’s survey weights, which scaled the individual OOP expenditures within each state to be representative of the national level. To estimate the net government subsidy (or net benefit), direct user fees, or individual OOP payments for each type of health service and health facility, were subtracted from the unit cost benefit of public spending on that service. Insurance reimbursement was also netted out. In such cases where the OOP payments exceeded the gross benefits of public subsidy, the calculation was truncated at zero for net benefits, since it would have yielded a negative number.

One significant data problem with OOP payments reported for public visits was that any subsequent private-sector visit related to that same illness episode would have been reported as an OOP payment associated with a public-facility visit. This probably resulted in an overestimate of OOP payments made solely for public visits. Furthermore, this result cannot be correctly estimated from these data. This is highlighted in several places in the discussion below. Similarly, subsequent OOP payments to public facilities after initial visits to private facilities for the same level of care were not included in the analysis. This may cancel out some of the overestimation.

### Socio-economic status

A measure of socio-econoimc status for each individual included in the analysis was needed to measure the benefits by income distribution. We utilized consumption expenditure data collected from the NSS survey. These data captured the consumption patterns over the last 30 days for each individual surveyed. Individual income was calculated by dividing household income by the number of individuals within the household. Consumption data were annualzed to 1 year and used to understand varying patterns of the utilization and benefits. For the purposes of this study, consumption was equated with income and proxied as a level of socio-economic status. In more recent studies, consumption expenditure has been seen as a more reliable measurement of wealth compared to income since it is easier to collect [15].

### BIA analysis

This BIA focuses on government funded health care facilities in India. The health facilities included in the analysis were public hospitals (national hospitals, regional/state hospitals, and other general hospitals), and public primary care facilities. In terms of service volume, as well as the financial value of public services received, service delivery benefits were estimated by service type, including inpatient admissions, outpatient contacts, and deliveries. These benefits were then distributed according to the income of the individuals receiving said benefits. To measure equity differences across geographic areas, the analysis was conducted both at the national level and at the individual state level.

Concentration curves were calculated for utilization of services, gross public benefits, and net benefits. Utilization concentration curves displayed the cumulative distribution of the population based on income level versus cumulative utilization of care. Net and gross benefit concentration curves displayed the cumulative distribution of the population based on income level versus the cumulative share of benefits accruing to that portion of the population. “Benefits received” were defined as either gross benefits (financial cost of each service type), or net benefits (cost of each service after removing insurance reimbursements and OOP expenditures). Concentration indices were estimated statistically and corresponded to twice the area between the concentration curve and the 45-degree line of perfect equality. Concentration indices ranging between 0 and 1 indicated pro-rich distributions, while concentration indices ranging between 0 and − 1 indicated pro-poor distributions. Indices were estimated both nationally and for all Indian states, as well as for each of the different service types. All concentration indices were conducted over both gross and net benefits. Concentration curves and concentration indices were estimated both nationally and at the state level. Stata was used to produce the BIA results. The Distributive Analysis Stata Package (DASP) was used to produce the concentration curves, and the *conindex* package was used to produce all concentration indices [16].

Each of the key indicators used to conduct the BIA were explained above and include measurements of socio-economic status, utilization rates,unit cost estimates, and benefits.

## Results

### Utilization: National level patterns in different types of services (inpatient, outpatient, delivery)

**Fig. 1:**
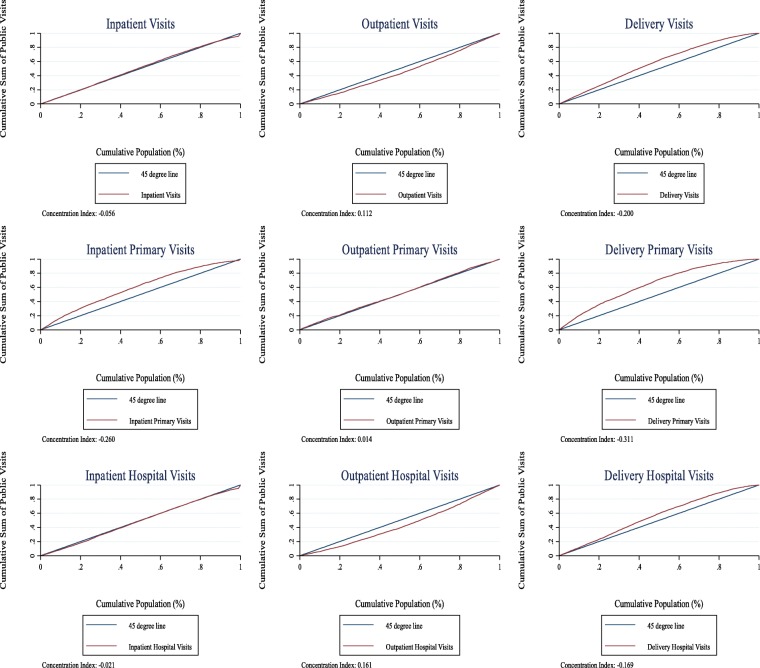
National-Level Public Sector Utilization by Sector & Care-Level

*Note*: Fig. 1 displays concentration curves for national-level inpatent, outpatient, and delivery public utilizaiton in row 1; national-level primary care inpatient, outpatient, and delivery utilization in row 2; and national-level hospital-level inpatient, outpatient, and delivery utilization in row 3. The curves reflect the cumulative sum of all public visits over the percentile of the population, ranked by income level. The concentration indices, calculated as the area between the curve and the 45 degree line of perfect equality, are presented below each graph. Negative concentration indices are relatively pro-poor while positive concentration indices imply a pro-rich distribution.

Figure 1 shows the concentration curves and the concentration indices for health care utilization for levels and types of services averaged across all states in India. The results show that both inpatient visits and deliveries demonstrate pro-poor patterns. Although deliveries are the most pro-poor with a concentration index of − 0.200, inpatient visit utilization is only slightly pro-poor with a concentration index of − 0.056. The utilization of outpatient visits is pro-rich with a concentration index of 0.112. The bottom two rows of Fig. 1 then show utilization by level of care (Level 1 (HSC), Level 2 (PHC, dispensary/CHC/mobile medical units), and Level 3 (public hospital)). The results show that inpatient pro-poor trends are driven mainly by inpatient visits at Level 1 and Level 2, while the outpatient pro-rich trends are driven by outpatient visits at public hopsitals.

### Benefit incidence: Gross versus net

**Fig. 2:**
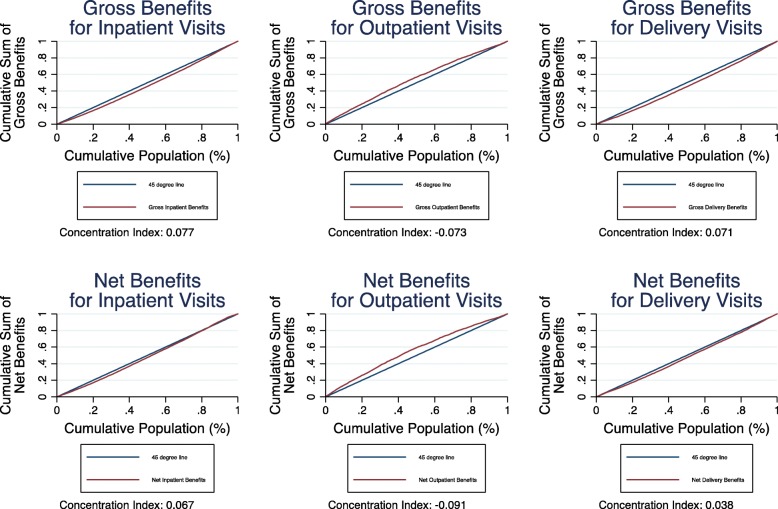
National-Level Public Benefits by Sector

Figure 2 displays charts for gross and net benefits. In general, when viewed from both a gross and net benefits perspective, public outpatient care is the most pro-poor service. Furthermore, inpatient and delivery services are distributed fairly close to the 45 degree line. There are some interesting changes between the distribution of health care utilization and that of gross and net benefits for different services in terms of inequality. Delivery services, which are distinctly pro-poor in utilization, are less so in gross and net benefits. Additionally, the utilization results in Fig. 1 show a pro-rich outpatient utilization pattern, yet reveal less inequality in the gross benefits. In contrast, Fig. 1 shows pro-poor inpatient and delivery patterns in utilization nationally, while Fig. 2 demonstrates greater equality in gross benefits, though slightly pro-rich. Further analysis is needed to determine if these differences are significant and what could explain them. Overall differences in utilization rates and costs across the states could account for these differences. For example, if wealthier individuals in larger, richer states are more likely to benefit from higher cost government inpatient and delivery services, this could result in more inequality in benefits than in utilization.

It is also interesting to note that gross and net benefits are relatively similar for all types of services. A large variation in OOP spending relative to unit cost for the wealthier would result in a larger difference in gross and net benefits, especially when compared against those same unit costs for the poor. The fact that gross and net benefits are relatively similar for all services suggests not only that almost everyone pays something OOP for services, but also that the distribution of those OOP expenses are fairly equal across income levels. The truncation of net benefits at zero discussed above may also mute any differences. Please see Additional file 1: Table S1 for a summary of concentration indices.

*Note*: Fig. 2 displays concentration curves for national-level public inpatent, outpatient, and delivery benefits. Gross benefits reflect state-level unit costs estimates applied to utilization, while net benefits reflect unit costs subtracting out-of-pocket expenditure. The curves reflect the cumulative sum of all public benefits over the percentile of the population, ranked by income level. The concentration indices, calculated as the area between the curve and the 45 degree line of perfect equality, are presented below each graph. Negative concentration indices are relatively pro-poor while positive concentration indices imply a pro-rich distribution.

### National averages contain substantial variations across Indian States

The large sample size of the NSS, as well as our state-level government health spending evidence enables estimation of the distribution of utilization and benefit-incidence at state level. Overall, the state-level results show that there are large variations across Indian states for both utilization patterns and benefit-incidence.

**Fig. 3:**
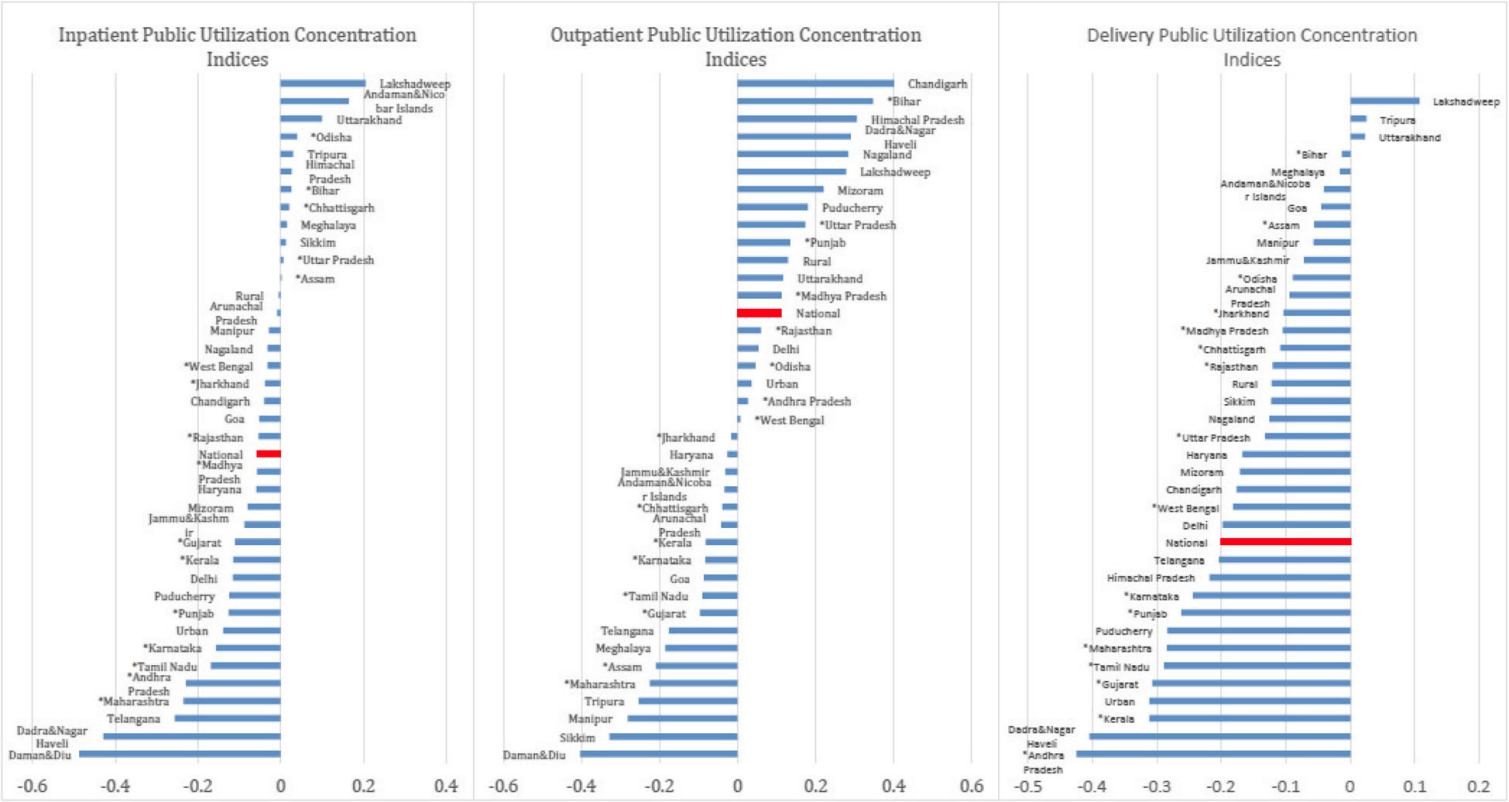
Public Utilization Concentration Indices by State and Sector

Note: Concentration Indices presented above are computed as the area between the concentration and the line of perfect equality for national-level inpatent, outpatient, and delivery public utilizaiton. Concentration curves reflect the cumulative sum of all public visits over the percentile of the population, ranked by income level. The concentration indices presented above are derived from separate curves for each state in India, broken down by sector. Negative concentration indices are relatively pro-poor while positive concentration indices imply a pro-rich distribution of utilization. National-level concentration indices, reflected directly in Fig. 1, are highlighted in red for comparison. Indian States with asterick represent states with direct unit cost calculation.

Figure 3 displays the concentration indices across states, arrayed from negative (pro-poor) to positive (pro-rich). For inpatient and deliveries in particular, seemingly equal national averages disguise large differentials across Indian states, despite the pro-poor utilization trends shown at the national level. This is especially evident for both public inpatient and outpatient care. For example, 62% of states exhibit pro-poor utilization trends for outpatient primary care, while only 25% do for outpatient hospital care. Additionally, 77% of states exhibit pro-poor benefit distributions for inpatient primary care in comparison to 60% for inpatient hospital care. When comparing inpatient and outpatient care, 69% of states are pro-poor for inpatient benefits in comparison to 54% for outpatient benefits and 91% for delivery benefits. While outpatient gross benefits are pro-poor for a number of Indian States, very few states show pro-poor net benefits for inpatient and delivery services.

**Fig. 4:**
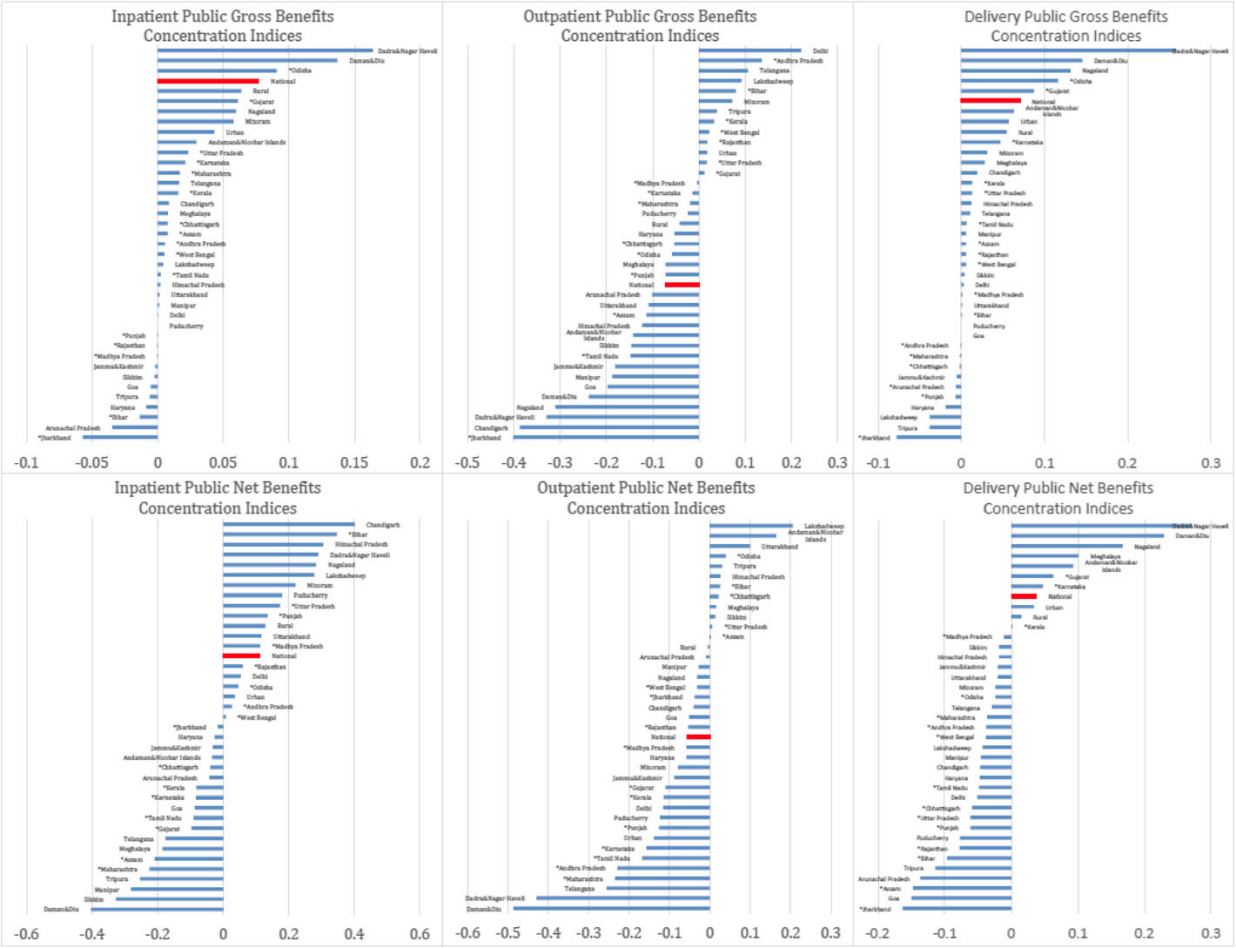
Gross and Net Benefits Concentration Indices by State and Sector

Note: Concentration Indices presented above are computed as the area between the concentration and the line of perfect equality for national-level inpatent, outpatient, and delivery public benefits. Concentration curves reflect the cumulative sum of all public benefits over the percentile of the population, ranked by income level. The concentration indices presented above are derived from separate curves for each state in India broken down by sector. In the top row, gross benefits are represented as unit costs applied to utilization by both state and sector, while in the second row, net benefits represent concentration indices for unit costs minus out-of-pocket expenditures. Negative concentration indices are relatively pro-poor while positive concentration indices imply a pro-rich distribution of benefits. National-level concentration indices, reflected directly in Fig. 2, are highlighted in red for comparison. Indian States with asterick represent states with direct unit cost calculation.

Figure 4 displays concentration indices for gross and net benefits across states, arrayed from negative (pro-poor) to positive (pro-rich). There is a considerable amount of variation across Indian states, despite a pro-poor trend shown at the national level for outpatient benefits and weakly pro-rich trends for inpatient and delivery care.

### Government service use and out-of-pocket spending

**Fig. 5:**
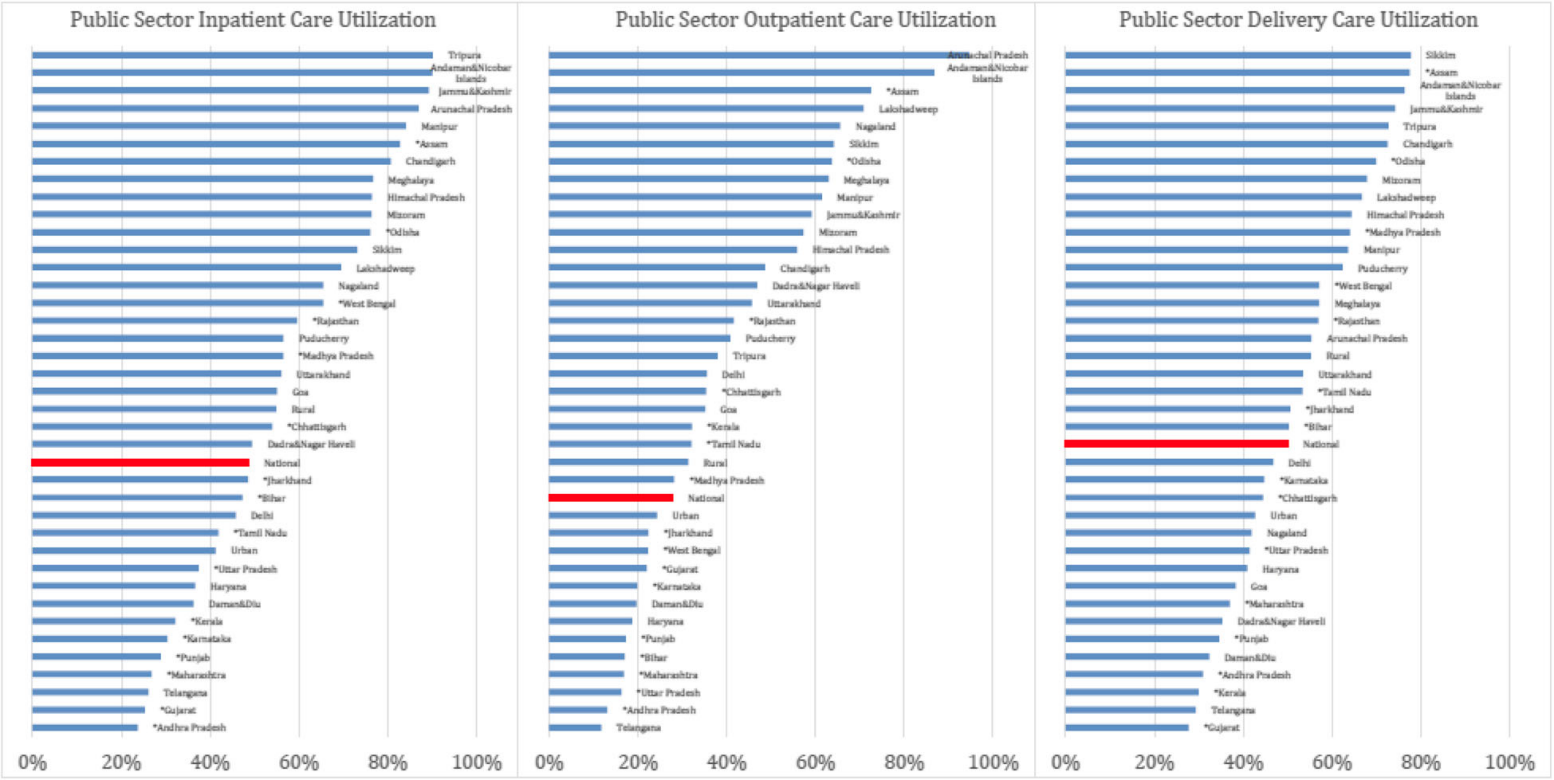
Percentage of Individuals Utilizing Public Sector Services by Sector and State

*Note:* The lines represent the population weighted percent of individuals receiving any type of care that utilizes public sector services (hospital and primary care combined). The results are broken down by sector: inpatient, outpatient, and delivery. The percentages represent every state, as well as urban, rural, and national aggregates. National aggeagates are highlighted in red for comparison. Indian States with asterick represent states with direct cost unit calculation.

Figure 5 displays the percent of individuals reporting use of government providers for different services, (inpatient, outpatient and deliveries) within each state. This varies considerably across states. The results showing use for different types of services, as well as hospital and primary care facility use, are combined. In general, individuals in smaller, more remote states depend more on government services, while those in larger states depend on government services less.

Table 1 shows a summary of OOP spending compared with government service unit costs for different types of services. The survey results demonstrate that, for all types of services, almost all of those who use government facilities report some level of OOP spending for that service. As noted above, this includes, but is not limited to, payments for health care covering prior episodes at locations other than government facilities. The NSS survey did not account for any subsequent private-sector OOP spending which followed other OOP spending on government services. Average OOP amounts vary by Indian state with the highest OOP spending for government services occurring in Manipur for outpatient, Himachal Pradesh for inpatient, and Kerala for deliveries.

**Table 1:** Out-of-Pocket Spending on Government and Private Services and Government Unit Costs for Specific Services

Type of Service	Type of Expenditure	Mean of Out-of-Pocket Expenditure per Visit and Public Unit Cost (Rupees)	Percent of Individuals Utilizing Care with Out-of-Pocket Expenditure
Outpatient	Total OOP	869.40	93%
	Public OOP	605.33	84%
	Private OOP	971.25	97%
	Unit Cost of Government Service	2472.92	–
Inpatient	Total OOP	17,081.91	99%
	Public OOP	6922.64	99%
	Private OOP	26,368.66	99%
	Unit Cost	7362.41	–
Delivery	Total OOP	5128.66	89%
	Public OOP	3067.05^a^	87%
	Private OOP	8371.54	98%
	Unit Cost Primary Level Facility	2472.92	–
	Unit Cost Hospital	7362.41	–

^a^ This is an average of primary OOP (2462.50) and hospital OOP (3221.30) for deliveries

These total service specific unit costs combining government service unit costs and associated OOP spending are also shown in Table 1. On average, the reported private sector OOP costs for outpatient visits alone (971 rupees) are 2107 rupees less than the average of the combined total of both the government unit cost for outpatient (2473 rupees), plus the OOP cost associated with use of government outpatient services (605 rupees). Similar results are present when comparing delivery services in public facilities to private delivery services. However for inpatient services, government unit costs plus the associated OOP costs are still much lower than the OOP costs of private hospitals. In addition, the results show that almost everyone in India who utilizes the private sector spends a considerable amount OOP to supplement government services.

It is important to note that these results are likely hiding large differences in the quality of care across different types of facilities, as well as between government and non-government providers. For example, non-governmental outpatient care providers also include large numbers of “less than fully qualified” providers [17]. More work is needed to understand the implications of these differences.

## Discussion

Several key findings emerge from this analysis. At the national level, utilization for public health facilities is pro-poor for both inpatient visits and deliveries, but slightly pro-rich for outpatients visits. When gross and net benefits are included in this analysis, public health spending approaches equality. However for all services, such spending still remains slightly pro-rich, while outpatient services trend slightly towards pro-poor. The discussion compares the results to other BIA analyses done globally and in India and also propose some possible causes and consequences of the findings.

Some of the results shown here are comparable to previous BIAs done in India. For example, Chakraborty et al. used data from both the 60th round and the 52nd round of the NSS to examine effectiveness of public health spending [12]. Their results show that for both inpatient and outpatient services, inequality in public health expenditure remains in favor of the rich [12]. A BIA of Northeast India by Ngangbam and Ladusingh, which used the 60th round of NSS data gathered in 2004, also showed that public health expenditure was pro-rich [13]. However, our data which uses the 70th round of the NSS shows more favorable equality in inpatient and delivery, as well as some pro-poor trends for outpatient. Similarly, other BIAs in developing countries depict public health expenditures as pro-rich. A systematic review of BIAs in 24 developing countries showed that benefits of health-care financing accrued more to the rich than the poor [5]. There are only a few places where BIAs have shown benefits in favor of the poor. A study done in Jordan in 2000 showed that the poorest quintile accounted for a greater proportion of public health subsidies, while another study done in Nigeria also demonstrated that the poor were the largest beneficiaries of net aggregates of benefits from priority public health services [7, 8].

With regard to the resuls shown above, it is interesting to note that utilization, with respect to delivery services, is the most pro-poor. One plausible cause of this trend could be some of the initiatives under the National Health Mission (NHM), which have reduced maternal and child health expenditures [6]. One such initiative is Janani Suraksha Yojana (JSY), implemented in 2005 to decrease disparities in access to institutional deliveries. Although inequities still persist, since the introduction of the program, levels of inequality have dropped and institutional deliveries have increased across all groups [18].

Although we see a more pro-poor trend in utilization for deliveries at the national level, when net benefits are included in the analysis, it becomes pro-rich. A potential cause of this trend is that, relative to the unit cost of services, poorer women are paying more OOP for the location where they decide to deliver. This is counterintuitive, as poorer women should theoretically qualify for the different incentive and reimbursement programs that are part of the NHM. Randive et al. postulate that these incentives are either insufficient, or that there are other factors accounting for some of this inequality, such as the higher male illiteracy rates or low-quality public health facilities in poorer areas [18]. A qualitative study by Vellakkal et al. also highlights several impediments to institutional delivery [19]. They note that, due to other associated costs (e.g. informal payments), the cash incentive component of JSY is not an enabling factor for institutional delivery in health facilities, and may actually cause poorer groups to opt out of utililizing such initiatives [19].

Looking beyond national averages, large variations among states can be seen with respect to both utilization and benefits. Pro-poor benefits are evident in a greater capacity for primary care services, than hospital services. Data from Chakraborty et al. suggest that there are consequences with regard to spending as a result of this inequality [12]. Their results reveal that states with higher percentages of inpatient services consumed by the wealthiest quintile often show regressive patterns of public health spending. Additionally, those states with pro-poor patterns of health spending in rural areas showed regressive patterns of health spending in urban areas [12]. Furthermore, national averages tend to mask variations within smaller geographical or local governmental areas, thus highlighting the need for disaggregated data to identify these differences.

Additionally, the results suggest consequences for utilization patterns by income levels and private and public sector. For example, with respect to all health services provided by the government health sector, some states show pro-poor utilization, and remain pro-poor when net benefits are considered. Maharashtra, Karnataka, and Tamil Nadu are some of those states. Conversely, states such as Uttarakkhand and Lakshadweep are pro-rich in utilization and remain so with respect to net benefits. A trend analysis by Acharya et al. comparing the two states of Tamil Nadu and Orissa from 1995 to 2004 showed that while public spending became more pro-poor across all services in Tamil Nadu, Orissa did not see those same trends [9]. Their analyses show that as the use of public services by the rich had declined considerably in Tamil Nadu, public spending had become more pro-poor, whereas in the case of Orissa, the rich were using more services than the poor. They postulate that the growing private health market in Tamil Nadu attracted the rich, which would justify this difference [9].

Despite the large dataset, as well as the inclusion of all Indian states in this anlaysis, there are several limitations with regard to the survey and the estimations. First, the design of the NSS does not allow the analyst to track the use of additional services or the payments for these services by each episode of illness. New rounds of the NSS need to take this into consideration in order to improve OOP esitmations for both public and private-sector use. Secondly, verification of estimated OOP payments and reimbursements need to be considered since about 11% of survey respondents reported reimbursements for services exceeding what they paid OOP. As described above, for these individuals, net benefits were classified as zero. Finally, due to costing studies on all types of services, the authors relied on an estimation of unit costs, which utilized both state-level government expenditures and reported utilizations from the NSS survey. Future analyses should take into consideration the updated costing data for relevant services. Regarding the BIA methodology, there are also several limitations, which should be highlighted to better rationalize and interpret these findings in relation to policy. These limitations include the fact that BIA equates expenditure with benefit and the that BIA analysis does not specify a model for the behaviors resulting in the observed distribution of benefits [20]. The first point is standard for most economic analyses utilizing expenditures but should be mentioned because if true experienced benefit per dollar is different depending on who receives that investment, in relation to their income than observed benefit inequities may divergere from these estimates. The direction of such a divergence is unclear and may be the topic of future research on benefit realization. Additionally, future work should be conducted to understand the behavioral patterns that underly these inequities in utilization and benefit to determine whether the root cause is poverty or another factor less readily quantifiable such as perceived quality, availability of private or traditional substitutes, or indirect costs. Finally, while BIA is useful to examine inequality across a continuum, it is more difficult to use the BIA to examine certain cohorts withing the continuum, such as the middle portion of the spectrum.

## Conclusion

Public health spending must be effectively allocated to address the health needs of the poor. This study set out to answer whether this was being done in India. Previous BIAs done in India have shown unequal distribution of resources with many revealing trends that benefit the rich. Although this analysis demonstrated many improvements India has made in reducing disparities, there is still much to do.

National-level results depict relative equality in the distribution of government spending for health services. This is a positive result, since in a mixed health system such as India’s, one should expect government health spending to be strongly pro-poor. However, further disaggregation of national results show wide variation in utilization rates and benefits among states, revealing that especially in rural areas, some states are doing far worse than others. India has significant information resources. Policy makers in India should use these data to expand the detail of their work and monitor levels and equity trends in government health spending, thereby improving the allocation of government resources to better target the disadvantaged.

## Additional file

Additional file 1: **Table S1.** Concentration Indices for Utilization, Gross Benefits and Net Benefits, by Level of Service. (DOCX 13 kb)

## Abbreviations

BIA: Benefit Incidence analysis
CHC: Community Health Centre
DASP: Distributive Analysis STATA Package
HSC: Health Sub Centre
INR: Indian Rupees
JSY: Janani Suraksha Yojana
NHM: National Health Mission
NSS: National Sample Survey
OOP: Out-Of-Pocket
PHC: Primary Helath Care
