# Correction to: benefit incidence analysis in public health facilities in India: utilization and benefits at the national and state levels

**DOI:** 10.1186/s12939-019-0945-y

**Published:** 2019-03-18

**Authors:** Diana Bowser, Bryan Patenaude, Manjiri Bhawalkar, Denizhan Duran, Peter Berman

**Affiliations:** 1 Heller School for Social Policy and Management, Brandeis University, 415 South Street, Waltham, MA 02453 USA; 2 Johns Hopkins Bloomberg School of Public Health, Baltimore, USA; 3 Harvard University T.H. Chan School of Public Health, Boston, USA

**Correction to: International Journal for Equity in Health 2019 18:13.**

**https://doi.org/10.1186/s12939-019-0921-6**

Following publication of the original article [1], the authors notified us of an error in the reported number of outpatient visits within the **Measuring health service utilisation** section. The correct number is 26 instead of 262 which was originally reported.

The original article was updated.
