# Supplementary material for: Benefit incidence analysis in public health facilities in India: utilization and benefits at the national and state levels
Source: Int J Equity Health. 2019 Jan 21;18:13. doi: 10.1186/s12939-019-0921-6 (PMC6341563; doi:10.1186/s12939-019-0921-6)
Supplement: Supplementary file 1 — Table S1. Concentration Indices for Utilization, Gross Benefits and Net Benefits, by Level of Service. (DOCX 13 kb) [file 12939_2019_921_MOESM1_ESM.docx]

**Supplemental Table:** Concentration Indices for Utilization, Gross Benefits and Net Benefits, by Level of Service

|  | **Inpatient** | **Outpatient** | **Delivery** |
| --- | --- | --- | --- |
| **Utilization** | -0.056 | 0.112 | -0.200 |
| **Gross Benefits** | 0.077 | -0.073 | 0.071 |
| **Net Benefits** | 0.067 | -0.091 | 0.038 |
